# Pole lengths influence O_2_-cost during double poling in highly trained cross-country skiers

**DOI:** 10.1007/s00421-017-3767-x

**Published:** 2017-12-01

**Authors:** Camilla Høivik Carlsen, Bjarne Rud, Håvard Myklebust, Thomas Losnegard

**Affiliations:** Department of Physical Performance, Norwegian School of Sport Sciences, Ullevål Stadion, Post Box 4014, 0806 Oslo, Norway

**Keywords:** Cross-country skiing, Exercise economy, Equipment, Center of mass, Skiing technique

## Abstract

**Purpose:**

In elite cross-country skiing, double poling is used in different terrain. This study compared O_2_-cost and kinematics during double poling with four different pole lengths [self-selected (SS), SS − 5 cm, SS + 5 cm, SS + 10 cm] at Low versus Moderate incline.

**Methods:**

Thirteen highly trained male cross-country skiers (mean ± SD 23 ± 3 years; 182 ± 4 cm; 77 ± 6 kg) completed eight submaximal trials with roller skis on a treadmill at two conditions: “Low incline” (1.7°; 4.5 m s^−1^) and “Moderate incline” (4.5°; 2.5 m s^−1^) with each of the four pole lengths. O_2_-cost and 3D body kinematics were assessed in each trial.

**Results:**

In Low incline, SS + 10 cm induced a lower O_2_-cost than all the other pole lengths [*P* < 0.05; effect size (ES) 0.5–0.8], whereas no differences were found between the remaining pole lengths (*P* > 0.05; ES 0.2–0.4). In Moderate incline, significant differences between all pole lengths were found for O_2_-cost, with SS − 5 cm > SS > SS + 5 cm > SS + 10 cm (*P* < 0.05; ES 0.6–1.8). The relative differences in O_2_-cost between SS and the other pole lengths were greater in Moderate incline than Low incline (SS − 5 cm; 1.5%, ES 0.8, SS + 5 cm; 1.3%, ES 1.0, and SS + 10 cm; 1.9%, ES 1.0, all *P* < 0.05). No difference was found in cycle, poling or reposition times between pole lengths. However, at both conditions a smaller total vertical displacement of center of mass was observed with SS + 10 cm compared to the other pole lengths.

**Conclusion:**

Increasing pole length from SS − 5 cm to SS + 10 cm during double poling induced lower O_2_-cost and this advantage was greater in Moderate compared to Low incline.

**Electronic supplementary material:**

The online version of this article (10.1007/s00421-017-3767-x) contains supplementary material, which is available to authorized users.

## Introduction

In classical cross-country skiing, double poling (DP) and diagonal stride (DIA) are the most frequent used sub-techniques in competitions. These sub-techniques are considered as a gearing system (Pellegrini et al. 2013), where DP traditionally was used in flat terrain and DIA preferred in uphills. However, skiers have now developed the technique and upper-body endurance and strength to also use DP during Moderate to steep uphill skiing. These sections are of special importance since ~ 50% of the total race time is spent in uphill terrain and is the major determinant of the overall performance during time trials (Andersson et al. 2010; Bolger et al. 2015; Sandbakk et al. 2016).

Skiing speed depends on several physiological and mechanical factors. One of these factors is the O_2_-cost of locomotion, defined as the amount of energy expended per unit of velocity (di Prampero 2003), and there have been reports of a close relationship between O_2_-cost and performance in cross-country skiing (Ainegren et al. 2013; Losnegard et al. 2017; Mahood et al. 2001). During DP, propulsive forces are transferred sorely through the poles, suggesting that pole length is an important parameter for O_2_-cost and performance (Losnegard et al. 2017). However, previous studies have exclusively investigated the influence of pole length in flat or slightly inclined terrain, i.e., < 2.5° (Hansen and Losnegard 2010; Hoffman et al. 1994; Losnegard et al. 2017; Nilsson et al. 2003, Onasch et al. 2016) and little is known about how pole length influences performance or performance-related mechanisms on steeper inclines.

The chosen pole length is a compromise between the optimal lengths used in different sub-techniques, exemplified by use of longer poles in ski skating (~ 90% of body height) than in classical style (traditionally between 82–85% of body height) (Hansen and Losnegard 2010). The reason for this difference is not clear, but according to anecdotes from the cross-country skiing milieu, the arm movement (“low shoulder”) during the reposition phase in DIA restricts the use of longer poles in classical style. However, it has been proposed that longer pole lengths in DP may have a greater advantage in uphill versus flat terrain (Losnegard et al. 2017) and thereby compensate for the possible disadvantages of DP in uphills compared to DIA. Therefore, from season 2016–2017, a temporary rule from the International Ski Federation (FIS) restricts the classical pole length to 83% (including ski boots, equivalent to ~ 85% of lean body height) (FIS 2017). FIS states that “the primary goal of this rule is not to ban double poling, but to add an additional tool to protect classical technique and all its aspects (diagonal, double poling, kick double poling, herringbone) so that competitions in classical technique are fair for everybody” (FIS 2016). However, scientific evidence of the effect of pole length in uphill terrain on performance or performance-related factors is limited.

During uphill DP, movement patterns change substantially compared to flat terrains (Millet et al. 1998; Pellegrini et al. 2013; Stöggl and Holmberg 2016). Skiers demonstrate a smaller hip flexion angle during the entire cycle, while flexion and extension of the knee and ankle joints are more pronounced with a greater range of motion (ROM) in uphill versus flat terrain (Stöggl and Holmberg 2016). In combination, these technical alterations enable the skier to reposition body segments and poles more rapidly for the next pole plant and allow the body mass to be more effectively used for the production of pole force (Holmberg et al. 2005; Stöggl and Holmberg 2016). However, such technical change could increase the moment of force in the knee and ankle joints, and thus require greater force production with subsequent higher energy consumption (Blanpied and Nawoczenski 2010). Recently, Losnegard et al. (2017) demonstrated that when DP with 7.5 cm longer poles in slightly inclined terrain (2.5°), skiers used more extended joints and a smaller ROM in the lower limbs compared to self-selected pole lengths. Since longer poles may cause a more upright working position and reduce the ROM in steeper terrain, longer poles may potentially have a greater impact on skiers’ O_2_-costs as inclination increases.

Considering the lower O_2_-cost induced by longer poles in slightly inclined terrain (Losnegard et al. 2017), together with the increasing use of DP in uphill, the present study investigated how pole length influences the O_2_-cost and joint kinematics during DP in two different uphill conditions. The main hypothesis was that longer poles would induce a lower O_2_-cost compared to self-selected pole lengths and that this difference would increase with steeper terrain.

## Methods

### Subjects

Thirteen highly trained male cross-country skiers (mean ± SD 23 ± 3 years; 182 ± 4 cm; 77 ± 6 kg) participated in the study. Their self-selected classic style pole length was 154 ± 3 cm (84 ± 1% of body height). Their maximal oxygen uptake, tested during treadmill running on a separate day, was 73 ± 3 mL kg^−1^ min^−1^ (range 68–77) [for the protocol see (Losnegard et al. 2014)]. The study was conducted according to the Declaration of Helsinki and to the Norwegian law. All the subjects gave their written informed consent before study participation.

### Experimental design

Eight of the 13 subjects had limited experience with roller skis on a treadmill prior to the project, and had therefore one familiarization session before taking part in the main tests. On the testing day, O_2_-cost and 3D body kinematics were recorded while roller skiing on the treadmill using DP. After 15 min warm-up (1.5°; 2.5–3 m s^−1^) at ~ 60–70% of maximal heart rate (HF_max_), the subjects completed two submaximal uphill conditions: “Low incline” (1.7° and 4.5 m s^−1^) and “Moderate incline” (4.5° and 2.5 m s^−1^). The speeds and inclines at each condition were matched for similar external power and were 199W ± 17 and 206W ± 17 in Low and Moderate incline, respectively. External power was calculated as the sum of the power against gravity and the power against rolling friction (Losnegard et al. 2013). Based on pilot testing, the inclines and speeds were chosen to induce a relevant DP technique and to obtain steady-state oxygen uptake. One condition consisted of four 5-min trials separated by 3-min breaks, one with each pole length. Each condition followed the same order (first: “Low incline”; second: “Moderate incline”), while the four pole lengths were counter-balanced in either increasing pole lengths (SS − 5 cm, SS, SS + 5 cm and SS + 10 cm) or (SS + 10 cm, SS + 5 cm, SS and SS − 5 cm). Pole lengths were 82 ± 1, 84 ± 1, 87 ± 1 and 90 ± 1% of body height, respectively, for SS − 5 cm, SS, SS + 5 cm and SS + 10 cm.

### Protocol and measurements

The O_2_-cost was determined as the average oxygen uptake (mL kg^−1^ min^−1^) from 3 to 4.5 min in each trial. Heart rate (beats min^−1^) was similarly averaged. Because of the O_2_-measurement apparatus, the subjects were unable to express their rating of perceived exertion [RPE; (Borg 1982)] during the trial. Therefore, at 4 min into the trial, they were asked to choose their RPE, which they then reported at the end of the trial. Gross efficiency was defined as the ratio between external power output (W) and aerobic energy turnover rate (W) and expressed relatively as a percentage (Losnegard et al. 2014).

**Fig. 1:**
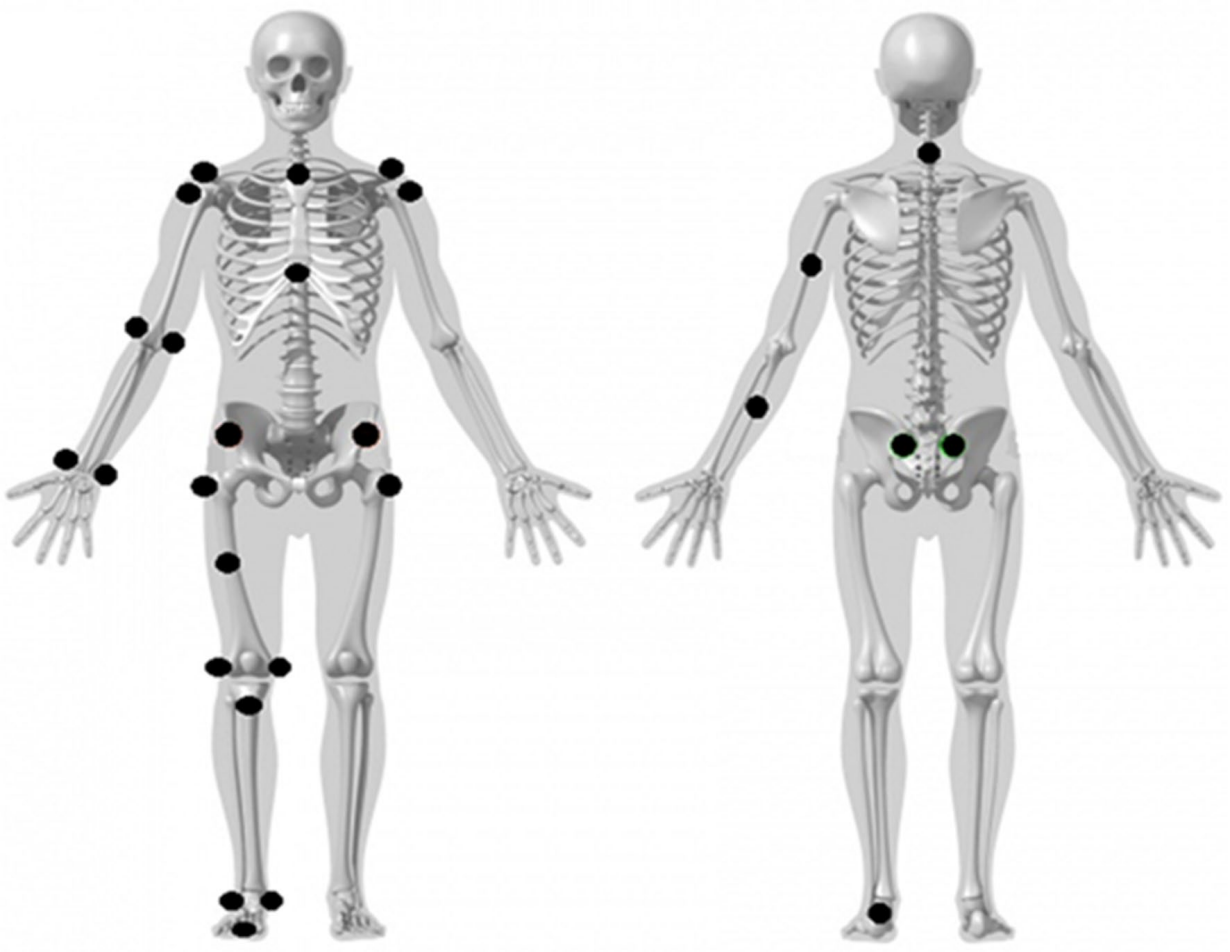
Illustration of the reflective marker placement. The segments are constructed by the following markers. Foot^a^: lat. malleolus, med. malleolus, calcaneus and 2. metatarsal head. Calf^a^: ant. tibial tuberositas. Thigh^a^: trochanter major^b^, ant. thigh, lat. and med. femoral epicondyle. Pelvis: ant. superior iliac spine^b^ and pos. superior iliac spine^b^. Truncus: acromion^b^, incisura jugularis, xiphoid process and cervical vertebra 7. Upper arm^a^: trochanter major humerus^b^, triceps, lat. and med. humerus epicondyle. Forearm^a^: ulna, process styloideus radii and ulnae. ^a^Right, ^b^right and left, *lat* lateral, *med* medial, *ant* anterior

Prior to each session, the motion capture system was calibrated following the manufacturer’s guidelines. Anthropometrical measurements of each subject (body height, length of leg, thorax, head plus neck and circumference of chest, right upper arm (proximal), elbow, wrist, thigh (proximal), knee- and ankle joint) were acquired. For construction of the modified 3D kinematic model, 27 reflective markers (spherical, 7 mm) were attached over the bony anatomical landmarks (pelvis, thorax, right upper and lower extremities) (Fig. 1). In addition, two markers were placed on the right pole (lateral aspect), 10 and 100 cm from the grip; two markers were placed on the right roller ski, in front of the rear wheel and behind the front wheel; and two markers were placed on the treadmill (85 cm apart) parallel to the skiing direction.

The 3D kinematics of the body, poles and roller skis were collected by a motion capture system in the last 30 s in each trial. The recording lasted 15 s with a sampling rate of 300 Hz. Before recording, the mouthpiece and sampling tube for the O_2_ measurements were removed without stopping the treadmill.

### Apparatus

All tests were performed on a treadmill (Rodby, Södertalje, Sweden). Prior, during and after the testing period, inclines and speed were controlled and did not show any changes. All subjects used the same pair of roller skis (Swenor Fibreglass, Swenor, Sarpsborg, Norway) with wheel types 2 (front) and 3 (rear). The roller skis had a friction coefficient of 0.026 µ and did not change during the testing period. The binding system was NNN (Rottefella, Klokkarstua, Norway). The subjects used Swix Triac 1.0 poles (Swix, Lillehammer, Norway) with a tip customized for treadmill roller skiing. Before the tests, the tips were adjusted to provide identical grip and weight.

Oxygen consumption was measured using an automatic ergospirometry system (Oxycon Pro, Jaeger GmbH, Hoechberg, Germany), as evaluated by Foss and Hallén (2005). Heart rate was measured with a Polar V800 (Polar Electro OY, Kempele, Finland). Anthropometrics were measured with a stadiometer (Seca 213, Hamburg, Germany) and measuring tape. Body mass (net mass and with equipment) was measured using a Seca scale (model 708, Hamburg, Germany).

Kinematic data were collected using a 3D motion capture system (ProReflex, Qualisys, Sävedalen, Sweden) with Qualisys Track Manager software (QTM) 2.7 and six cameras (Oqus 4, Qualisys Medical AM, Göteborg, Sweden). The global coordinate system was defined as follows: the incline of the treadmill was set to 0°; the *x*-axis was the longitudinal axis of the treadmill (the direction of motion); the *y*-axis was the side-to-side direction across the treadmill; and the *z*-axis was perpendicular to the ground. Visual 3D (C-motion, Inc., USA) and MATLAB (MathWorks, Inc., Natick, MA, USA) were used for further analysis.

### Data analysis

**Fig. 2:**
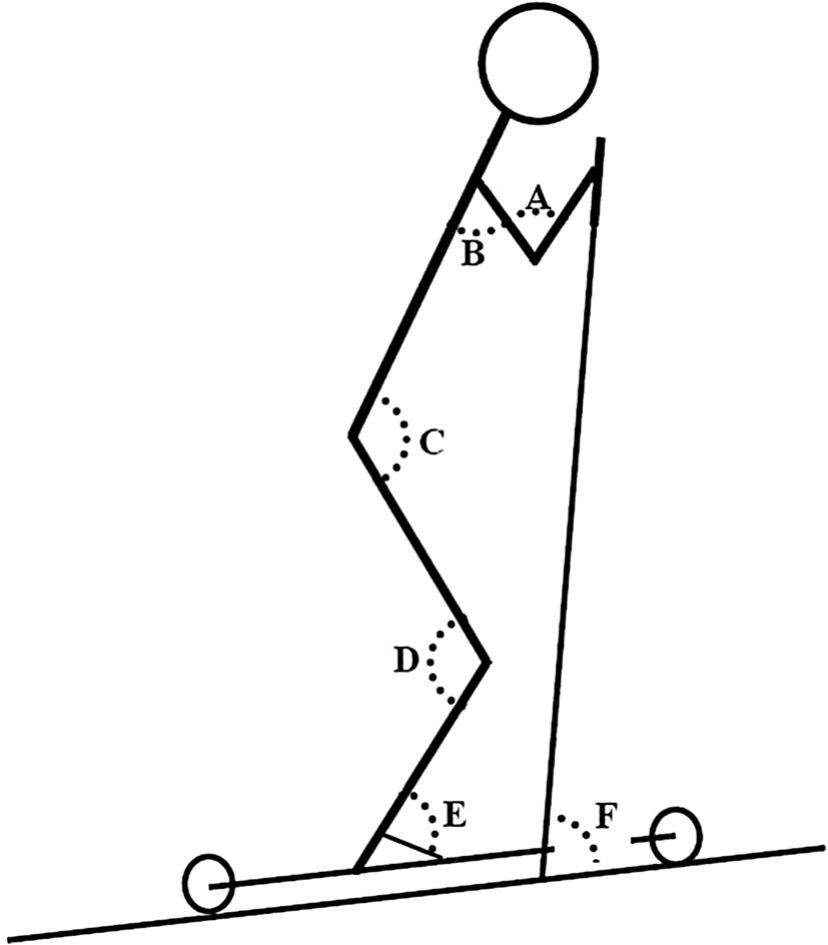
Illustration of the examined joint and pole angles. A = elbow, B = shoulder (sagittal and frontal plan), C = hip, D = knee, E = ankle and F = pole. For the hip, knee and elbow joints, the maximal extension was defined as 180°. For the ankle joint, 180° represented maximal plantar flexion. For the shoulder joint, the neutral position was defined as 0°. In the sagittal plane, angles > 0° indicated flexion and angles < 0° indicated extension and in the frontal plane full abduction was defined as 180°. For the pole, 0° indicated a right angle between the pole and the horizontal plane

Kinematic raw data were filtered (4th order butterworth low-pass filter, cutoff frequency of 6 Hz) and further processed in Visual3D and MATLAB. A kinematic 3D model of the thorax, pelvis, right arm and leg, together with the right pole and ski was created. Centers of the examined joints were found from the reflective markers (Fig. 1). Cycle time was defined as the time between two pole plants, poling time as the time between pole plant and subsequent pole liftoff, and reposition time as the time between pole liftoff and subsequent pole plant. Pole plant and pole liftoff were determined from the path of the pole markers in Visual 3D, where the pole plant was determined as the maximum forward position in the horizontal plane and pole liftoff was determined as the minimum vertical value in the sagittal plane. The pole angle relative to the treadmill belt plane at pole plant (pole angle_pole plant_) and (pole angle_pole liftoff_) were calculated in Visual3D. Illustrations of the examined joint angles are provided in Fig. 2.

The vertical position of the center of mass (COM_z_) was derived from seven body segments (forearm including the hand, upper arm, trunk and head, pelvis, thigh, leg, and foot), together with two segments for ski and pole. The relative mass of each body segment with respect to the total body mass was calculated based on De Leva (1996). As double poling consists of more or less synchronous movement patterns for the right and left limbs, the 3D model was constructed by extrapolating the right body segment data to also represent the left side segments. The equipment was weighed independently, and the weights of the ski boots were added to the foot segment. Each body segment’s COM was calculated with respect to its proximal segmental reference (De Leva 1996), and the COM for the whole body plus equipment was calculated.

For each condition, joint angles and COM_z_ were calculated from five consecutive cycles. For comparison, each cycle was time normalized using a third-order 101 point interpolation. The average over five consecutive cycles was used for statistical comparison. Joint angles and the vertical position of COM_z_ for each pole length were compared at pole plant, pole liftoff, and maximum (max) and minimum (min) values during the cycle. The vertical displacement of COM_z_ was calculated from the maximal and minimum values of the vertical position of COM_z_ (COM_zmax_ – COM_zmin_), regardless of when in the cycle it appeared. Ankle joint coordinates at the pole plant was used as reference point to assess the relative placement of the pole tip. Reported values, *D*_forward-poleplant_ and *D*_backwards-poleliftoff,_ are the distance between this reference point and the pole tip at the pole plant and pole liftoff, respectively. The shortest distance between COM and the poles at the pole plant (*D*_COM-poleplant_) were calculated from COM_z_ and the coordinates of the pole markers.

### Statistical analysis

Raw data were checked for normal distribution (Shapiro–Wilks) and presented as mean ± standard deviation (SD) if not otherwise stated. Relative differences between pole length and conditions are presented as mean ± 95% confidence interval (CI). Initially, the main effects of pole length and inclination, as well as the interaction between pole lengths and inclination, were checked with a two-factor within-subject ANOVA (4 × 2 design). If a main effect of pole length was found, one-way ANOVA comparing pole lengths for each of the two inclinations were conducted separately, followed by a Bonferroni post hoc correction for multiple comparisons. If a main effect of inclination was found, paired-samples *T* test was used to compare Low incline and Moderate incline inwardly for one pole length separately.The magnitude of the difference between pole lengths was expressed as standardized mean differences [Cohen’s *d* effect size (ES)]. The criteria for interpreting the magnitude of the ES were classified as trivial 0.0–0.2, small 0.2–0.6, moderate 0.6–1.2, large 1.2–2.0 and very large > 2.0 (Hopkins et al. 2009). Statistical calculations were performed using Microsoft Office Excel 2010 (Microsoft, Redmond, USA) and IBM SPSS Statistics 20.0 (International Business Machines (IBM), New York, USA). The level of confidence was set to 95% and a *P* value ≤ 0.05 was considered statistically significant.

## Results

### O_2_-cost

**Fig. 3:**
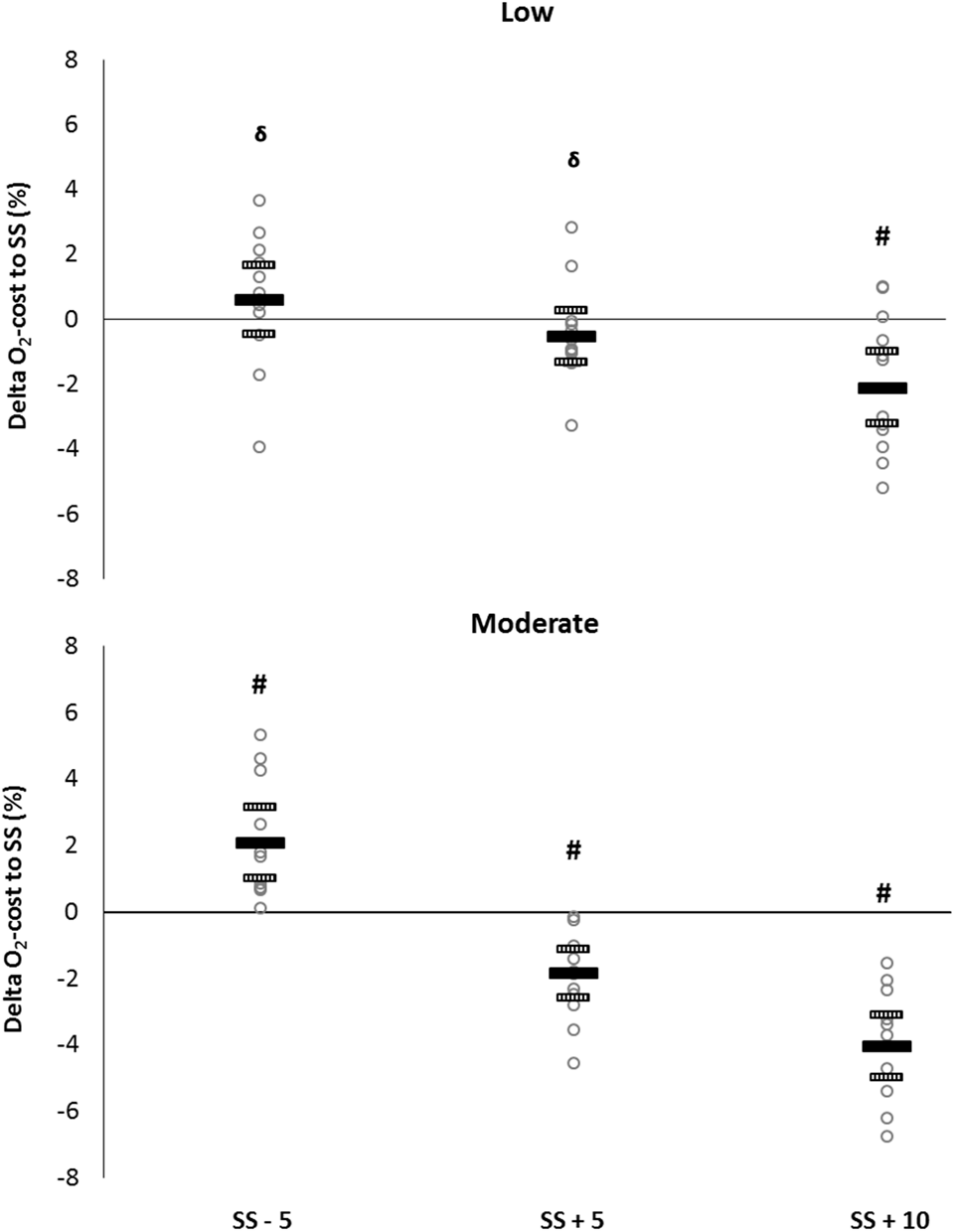
Individual differences in O_2_-cost relative to self-selected pole length in Low incline (1.7°; upper panel) and Moderate incline (4.5°; lower panel). Data are presented as individual (gray circles) and mean (black line) with 95% CI (dotted line). ^δ^Significant difference from SS + 10 cm (*P* < 0.05). ^#^Significant difference between all pole lengths (*P* < 0.05)

There was a significant main effect of pole length [*F*(3,36) = 31.6, *P* < 0.05], inclination [*F*(1,12) = 186.6, *P* < 0.05] and of the interaction between pole lengths and inclination [*F*(3,36) = 10.9, *P* < 0.05] on O_2_-cost. In Low incline, SS + 10 cm had a lower O_2_-cost than SS − 5 cm, SS and SS + 5 cm (*P* < 0.05; ES 0.5–0.8). No significant differences between SS − 5 cm, SS and SS + 5 cm were found (*P* > 0.05; ES 0.2–0.4). In Moderate incline, there was a significant difference between all four pole lengths, with SS + 10 cm inducing the lowest O_2_-cost compared to the other pole lengths (SS − 5 cm > SS > SS + 5 cm > SS + 10 cm; *P* < 0.05; ES 0.6–1.8) (Table 1). In Low incline, the relative difference (± CI) in O_2_-cost between SS + 10 cm and SS was − 2.1 ± 1.1% (*P* < 0.05; ES 0.6). In Moderate incline, the relative difference were 2.1 ± 1.1% (*P* < 0.05; ES 0.6), − 1.9 ± 0.7% (*P* < 0.05; ES 0.6) and − 4.0 ± 1.0% (*P* < 0.05; ES 1.5), respectively, for SS − 5 cm, SS + 5 cm and SS + 10 cm (Fig. 3). The relative difference in O_2_-cost between SS and the pole lengths was greater in Moderate incline compared to Low incline for SS − 5 cm (1.5%, ES 0.8), SS + 5 cm (1.3%, ES 1.0) and SS + 10 cm (1.9%, ES 1.0), all *P* < 0.05. There was no main effect of pole length on heart rate [*F*(3,36) = 3.6, *P* > 0.05], but there was a significant effect of inclination [*F*(1,12) = 27.7, *P* < 0.05] and of the interaction between pole lengths and inclination [*F*(3,36) = 6.3, *P* < 0.05]. The relative difference in heart rate between SS and pole lengths was only significant for SS − 5 cm and was greater in Low incline compared to Moderate incline (− 1.7%, ES 1.3, *P* < 0.05). Pole length [*F*(3,36) = 3.3, *P* > 0.05] and the interaction between pole lengths and inclination [*F*(3,36) = 0.4, *P* > 0.05] had no main effect on RPE, but inclination had a significant effect [*F*(1,12) = 24.5, *P* < 0.05]. The relative difference of RPE between SS and pole lengths was only significant for SS − 5 cm and was greater in Low incline compared to Moderate incline (2.0%, ES 0.4, *P* < 0.05) (Table 1).

**Table 1:** Physiological responses (upper part), and temporal and kinematic characteristics (lower part) for all pole lengths in Low and Moderate incline

Variable	Incline	SS − 5 cm	SS	SS + 5 cm	SS + 10 cm
O_2_-cost (mL kg^−1^ min^−1^)	Low	46.1 ± 1.5	45.8 ± 1.7	45.5 ± 1.4	44.8 ± 1.6^*,",δ^
	Moderate	50.0 ± 2.2	48.9 ± 1.5^*^	48.0 ± 1.6^*^	46.9 ± 1.1^*,",δ^
Heart rate (beats min^− 1^)	Low	149 ± 10	151 ± 9	150 ± 9	148 ± 8
	Moderate	155 ± 11	154 ± 10	153 ± 10	152 ± 10
Gross efficiency (%)	Low	16.2 ± 0.6	16.3 ± 0.7	16.4 ± 0.6^*^	16.6 ± 0.7^*^
	Moderate	15.6 ± 0.8	15.9 ± 0.6^*^	16.2 ± 0.6^*,"^	16.6 ± 0.5^*,",δ^
RPE (6–20)	Low	12.6 ± 1.8	12.2 ± 1.8	12.2 ± 1.4	12.3 ± 1.3
	Moderate	13.7 ± 1.6	13.5 ± 1.6	13.3 ± 1.4	13.3 ± 1.2
Cycle time (s)	Low	1.16 ± 0.08	1.19 ± 0.11	1.17 ± 0.09	1.17 ± 0.10
	Moderate	1.09 ± 0.08	1.11 ± 0.14	1.14 ± 0.10	1.13 ± 0.09
Poling time (s)	Low	0.37 ± 0.04	0.38 ± 0.06	0.38 ± 0.06	0.37 ± 0.05
	Moderate	0.49 ± 0.05	0.50 ± 0.05	0.51 ± 0.06	0.51 ± 0.06
Reposition time (s)	Low	0.79 ± 0.06	0.80 ± 0.07	0.79 ± 0.07	0.80 ± 0.08
	Moderate	0.60 ± 0.06	0.61 ± 0.06	0.61 ± 0.05	0.61 ± 0.06
D_forward-pole plant_ (cm)	Low	52 ± 16	53 ± 19	49 ± 16	45 ± 18^*,"^
	Moderate	33 ± 12	28 ± 15	26 ± 18	24 ± 15^*^
D_backwards-pole liftoff_ (cm)	Low	− 112 ± 5	− 118 ± 10^*^	− 121 ± 10^*^	− 124 ± 7^*^
	Moderate	− 90 ± 6	− 97 ± 8^*^	− 101 ± 10^*,′′^	− 103 ± 6^*^
Pole angle_pole plant_(˚)	Low	81 ± 7	81 ± 8	81 ± 7	81 ± 7
	Moderate	73 ± 5	73 ± 6	74 ± 7	73 ± 6
Pole angle_pole liftoff_ (˚)	Low	29 ± 2	28 ± 2	28 ± 2	28 ± 2
	Moderate	31 ± 3	31 ± 2	31 ± 2	30 ± 3
COM_z_displacement (cm)	Low	20 ± 2	20 ± 2	19 ± 3^*^	19 ± 2^*,′′^
	Moderate	21 ± 2	20 ± 2^*^	20 ± 3^*^	19 ± 2^*^
D_COM-pole plant_(cm)	Low	0.45 ± 0.05	0.45 ± 0.05	0.43 ± 0.05	0.42 ± 0.06
	Moderate	0.38 ± 0.03	0.36 ± 0.04	0.35 ± 0.05	0.33 ± 0.03^*,′′^

Data are presented as mean ± SD (*n* = 13)

*RPE* rate of perceived exertion, *D*_*forward-poleplant*_ *andD*_*backwards-poleliftoff*_ *(cm)* distance from the pole tip to the ankle joint, *Pole angle*_*poleplant*_ *and pole angle*_*poleliftoff*_ *(˚)* pole angle relative to the treadmill belt plane, *COM*_*z*_ the vertical center of mass, *D*_*COM-poleplant*_ *(cm)* the shortest distance between COM and the poles at the pole plant

*Significant difference from SS − 5 cm (*P* < 0.05)

′′Significant difference from SS (*P* < 0.05)

^δ^Significant difference from SS + 5 cm (*P* < 0.05)

### Displacement of COM

**Fig. 4:**
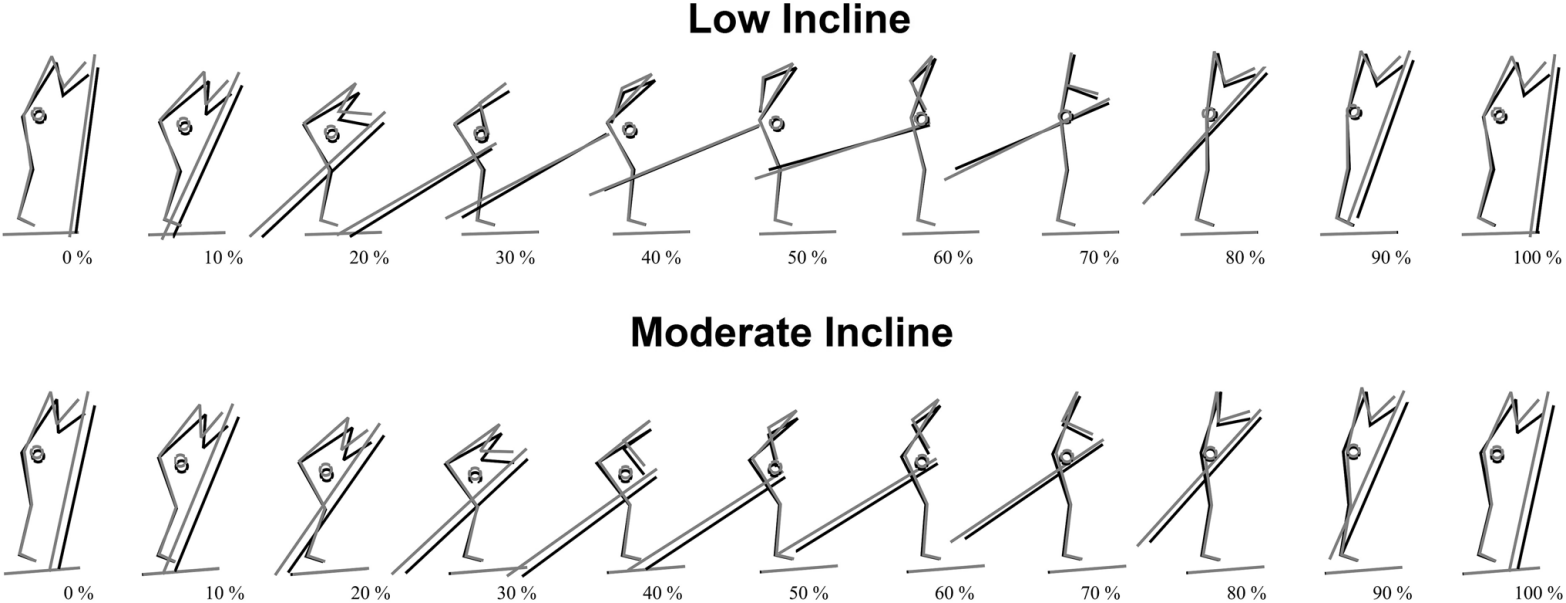
Kinematic illustration of a full double poling cycle between two pole plants (0–100%). A comparison between SS − 5 cm (black) and SS + 10 cm (grey) in Low incline (upper panel) and SS − 5 cm (black) and SS + 10 cm (grey) in Moderate incline (lower panel). Circles indicate the center of mass. Data are mean values, *n* = 13. For video animation of the above-mentioned comparisons see Online Resources 1 and 2

**Fig. 5:**
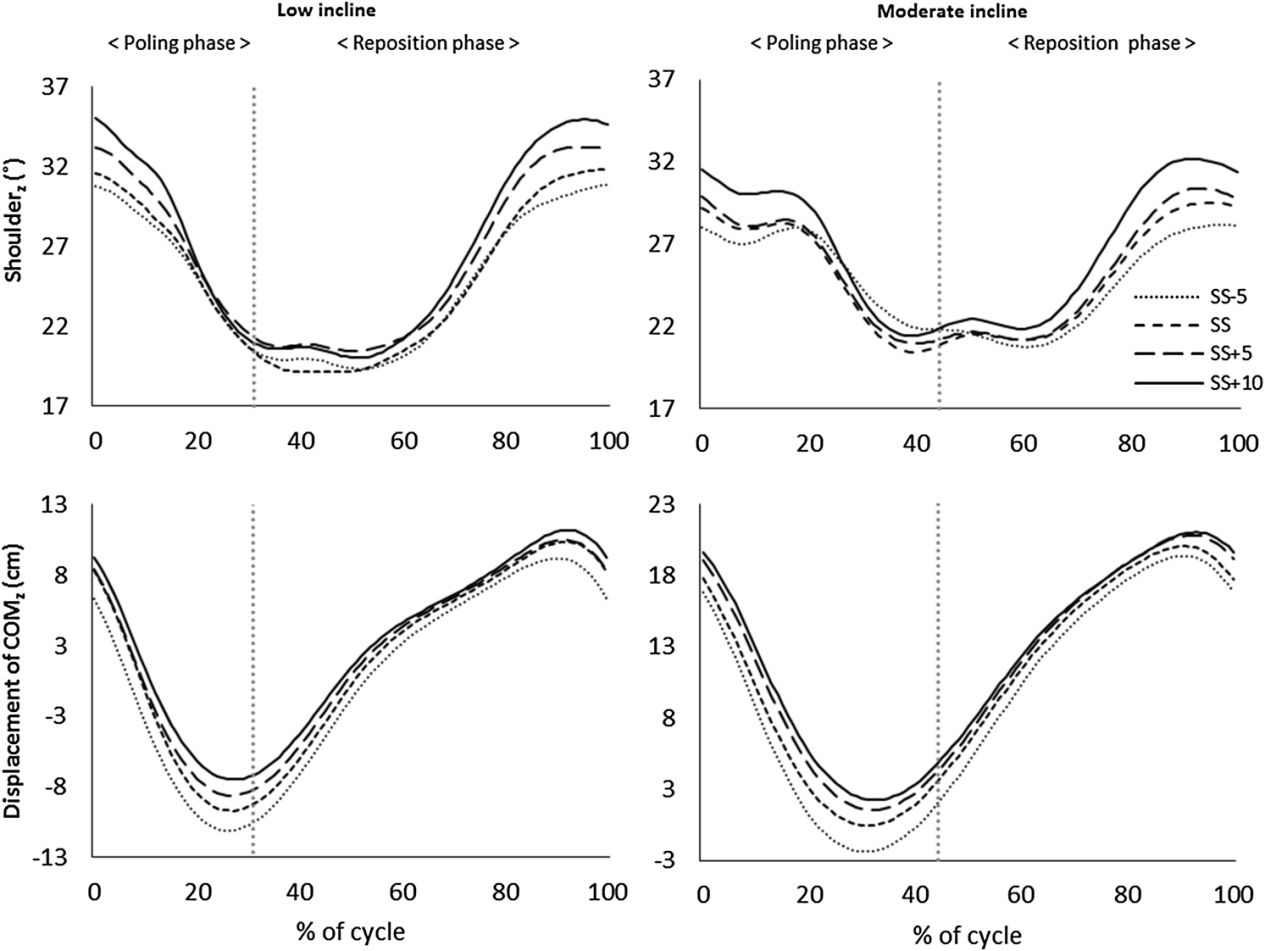
Shoulder abduction angle (˚) (upper panel) and vertical displacement of COM_z_ (cm) (bottom panel) during a full cycle in Low incline (1.7°; left) and Moderate incline (4.5°; right) for the range of pole lengths. The cycle starts (0%) and ends (100%) at the pole plant, and is divided into the poling and reposition phases (vertical dotted line). Data are mean values, *n* = 13

There was a significant main effect of pole length [*F*(3,33) = 16.4, *P* < 0.05] and of inclination [*F*(1,11) = 6.6, *P* < 0.05] on the total displacement of COM_z_. However, no significant interaction was found between pole lengths and inclination [*F*(3,33) = 2.1, *P* > 0.05]. Post hoc analyses showed that SS − 5 cm had the largest total displacement of COM_z_ and was significantly different from SS + 5 cm and SS + 10 cm in Low incline, and different from all other pole lengths in Moderate incline (*P* < 0.05; ES 0.6–1.0) (Table 1). However, the total displacement of COM_z_ when pole length increased from SS − 5 cm to SS to SS + 5 cm to SS + 10 cm (SS − 5cm to SS + 10 cm) was almost identical between Low and Moderate incline (Figs. 4, 5; Table 1).

### Joint kinematics

**Fig. 6:**
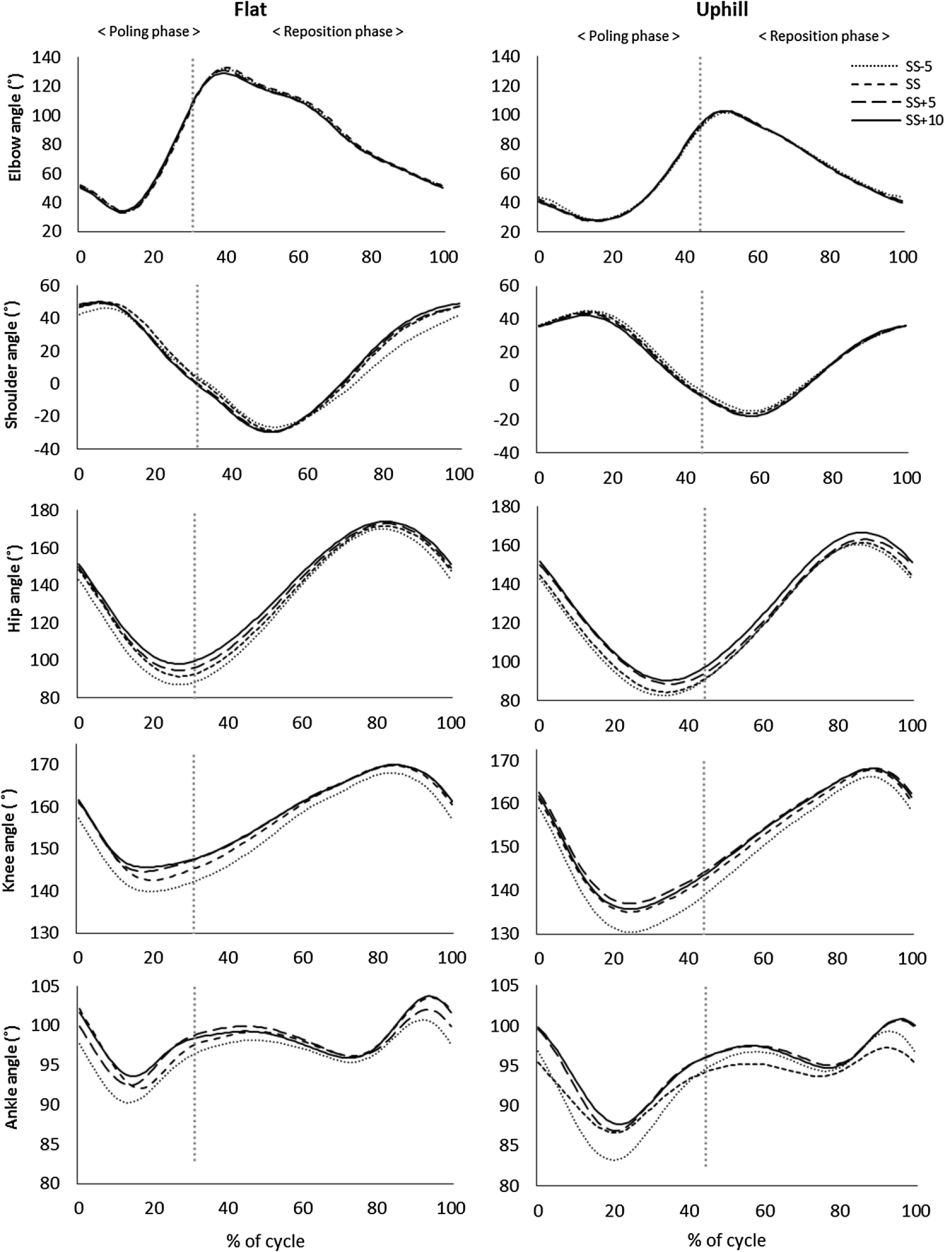
Elbow, shoulder (flexion/extension), hip, knee and ankle angles (˚) during a full cycle in Low incline (1.7°; left) and Moderate incline (4.5°; right) for the range of pole lengths. The cycle starts (0%) and ends (100%) at the pole plant, and is divided into poling and reposition phases (vertical dotted line). Data are mean values, *n* = 13

There were a significant main effect of pole lengths on hip, knee and ankle angle in both Low and Moderate inclines (all, *P* < 0.05). The hip and knee joints were more extended at the pole plant, pole liftoff, and minimum and maximum angles with SS + 10 cm than with SS + 5 cm, SS or SS − 5 cm in both Low and Moderate incline, but in Moderate incline the knee had the largest extension with SS + 5 cm. During the DP cycle, the ROM for the hip and knee joint decreased with increasing pole length from SS − 5 cm to SS + 10 cm in both Low and Moderate inclines, but in Moderate incline the knee joint had the smallest ROM with SS + 5 cm (Figs. 4, 6).

There was a significant main effect of pole lengths on shoulder abduction angle at the pole plant in both the Low and Moderate inclines (*P* < 0.05). Shoulder abduction at the pole plant increased with increasing pole length from SS − 5 cm to SS + 10 cm in both Low and Moderate inclines, but there was only a significant difference between SS + 10 cm and SS − 5 cm and SS (both, *P* < 0.05) in the Low incline trials (Fig. 5).

There was a significant main effect of pole lengths on elbow angle at pole plant in Moderate incline (*P* < 0.05), but not in Low incline (*P* > 0.05). Elbow flexion at the pole plant increased with longer pole length, but there was only a significant difference between SS + 10 cm and SS − 5 cm, and SS + 5 cm and SS − 5 cm (*P* < 0.05) (Fig. 6).

There was a non-significant main effect of pole lengths on pole angle relative to the horizontal in both Low and Moderate inclines (*P* > 0.05). However, there was a significant main effect of pole length on the distance between the pole tip and the ankle joint at the pole plant (*P* < 0.05) for both Low and Moderate inclines. SS + 10 cm showed a pole plant closer to the ankle joint compared to SS − 5 cm and SS in Low incline (*P* < 0.05) and SS − 5 cm in Moderate incline (*P* < 0.05) (Table 1). Thus, the distance between the COM and the pole (*D*_COM-poleplant_) was less with SS + 10 cm in Moderate incline (*P* < 0.05). The relative difference in *D*_COM-poleplant_ between SS + 10 cm and SS − 5 cm was greater in Moderate incline compared to Low incline (Table 1).

No significant main effects of pole length on cycle time, poling time or reposition time were found in either Low or Moderate incline (*F*_3,36_ = 0.6–2.1, *P* > 0.05); however, the poling and reposition times for each pole length showed large differences when Low and Moderate inclines were compared (all, *P* < 0.05) (Table 1).

## Discussion

This study investigated how pole length affects O_2_-cost and joint kinematics during DP while treadmill roller skiing in two different uphill conditions. The main findings were that increasing pole length from SS − 5 cm to SS + 10 cm (I) induced a lower O_2_-cost, (II) with a greater advantage in Moderate incline compared to Low incline and (III) resulted in a more upright posture with reduced total displacement of COM_z_ during the DP cycle.

The study demonstrated the relationship between pole length and O_2_-cost, showing that longer poles induced a lower O_2_-cost compared with shorter and self-selected pole lengths. These results are in accordance with Losnegard et al. (2017) and Onasch et al. (2016), and imply that a pole length up to at least ~ 90% of body height reduces the O_2_-cost during DP on a treadmill. Notably, these studies have exclusively tested male skiers, and the influence of pole length on O_2_-cost during DP for female skiers is currently not known.

In Low incline, the O_2_-cost was 2% lower with SS + 10 cm compared to SS, while in Moderate incline a 4% difference was found. Together with the findings from Losnegard et al. (2017), where a 2.5% lower O_2_-cost with SS + 7.5 cm compared to SS was found at 2.5°, the present data suggest that the advantage of longer poles increases with the steepness of the incline. Considering that ~ 50% of total race time is spent in the uphill terrain (Andersson et al. 2010; Bolger et al. 2015), and that uphill performance correlates most strongly with overall performance (Sandbakk et al. 2016), the choice of pole length could potentially have a significant impact on cross-country skiers’ performance. However, whether our findings can be transferred to snow conditions needs to be examined, in addition to the effects of skiing at high speeds, which are not well documented. These aspects are presently the subject of debate, since FIS (FIS 2017) recently introduced a restriction on pole lengths longer than 83% of body height (including ski boots) in classic-style skiing competitions.

The flexion–extension pattern in the lower limbs was independent of pole length (Fig. 6), but longer poles resulted in a more upright posture caused by more extended ankle, knee and hip joints, with the hip as the most pronounced extension compared to the knee and ankle joints (Fig. 4). Consequently, the total displacement of COM_z_ was reduced with longer poles. Taken together with previous results (Losnegard et al. 2017), it seems that the reduced overall displacement of COM_z_ contributes to reducing the O_2_-cost during DP. However, the relative difference in COM_z_ displacement from SS − 5 cm to SS + 10 cm in Low and Moderate inclines was almost identical (Table 1), suggesting that COM_z_ displacement alone does not explain why longer poles reduced the O_2_-cost more in Moderate incline compared to Low incline. In addition, the present study was conducted on an indoor roller ski treadmill with no aerodynamic drag. Hence, the potential negative effects of the more upright posture on drag forces with longer poles should be considered, particularly at high skiing speeds, when transferring the results to outdoor skiing.

In general, as the steepness of the uphill increases, the amount of work against gravity will also increase. To maintain the same external power, the speed must be reduced and, subsequently, poling time will increase (Losnegard et al. 2017; Stöggl et al. 2011). The poling time affects the muscle contraction time, which according to Hill’s law (Hill 1938) influences the effectiveness of muscle contractions. Therefore, the difference in O_2_-cost between pole lengths in Low incline versus Moderate incline could be influenced by the different speeds. However, Losnegard et al. (2017) did not find a statistical interaction between the pole length and speed on O_2_-cost within a range of speeds from 3.0 to 4.0 m s^−1^. The speeds in the present study were only slightly outside this range (2.5 and 4.5 m s^−1^), and we believe that the difference in O_2_-cost between pole lengths in Low incline compared to Moderate incline cannot fully be explained by the differences in speed in Low and Moderate inclines.

During DP, increasing pole length from SS − 5 cm to SS + 10 cm caused a more upright working position due to reduced flexion in the hip before pole plant in both Low and Moderate inclines. This technical alteration reduced the distance between the COM and the poles (*D*_COM-poleplant_), and led to a pole plant closer to the ankle joints in both Low and Moderate inclines (Table 1). Interestingly, as for O_2_-cost, the difference in *D*_COM-poleplant_ between SS − 5 cm and SS + 10 cm was more pronounced in Moderate incline compared to Low incline. This could cause a smaller external moment arm and torque in the working joints and further result in a better working posture, so the same amount of work would be maintained with a lower O_2_-cost. Further, DP in different terrains demands clear differences in movement patterns to overcome the external force. With increasing speed during DP in flat terrain, a short poling time is a limiting factor and emphasizes the need for high peak forces generated during a short period of time (Holmberg et al. 2005; Stöggl and Holmberg 2011, 2016; Stöggl et al. 2011). Elite skiers with higher impulse of resultant and horizontal pole force and longer time to peak pole force showed a distinct “pre-preparation” approach with a more forward pole plant (Stöggl and Holmberg 2011), and thus increased *D*_COM-poleplant_ to gain sufficient time to provoke a pre-activation of muscles before peak pole forces occur. Unpublished data from our laboratory indicates that pole lengths 90% of body height are not more beneficial at high speeds (8–10 m s^−1^) compared to self-selected poles (84% of body height), which could, to some extent, be related to the mechanisms mentioned above. As poling time does not seem like a limiting factor in uphill skiing, but rather work against gravity and the ability to use the lowered COM, potentially with a small *D*_COM-poleplant,_ could together with the above-mentioned explain some of the greater effect on O_2_-cost of increased pole length in Moderate incline compared to Low incline. However, future studies should include kinetic measurements to investigate these assumptions.

A novel finding from the present study was that the shoulders became more abducted at the pole plant in both Low and Moderate incline roller skiing when pole length increased. More abducted shoulders together with smaller elbow angles (Figs. 5, 6) have previously been characterized as a “wide elbow” DP strategy, which is described by specific pole force and muscle activity characteristics directly correlated to DP velocity (Holmberg et al. 2005). Hence, together with more extended ankle, knee and hip joints, more abducted shoulders resulting from longer pole lengths could be a strategy for DP economy enhancement.

## Conclusion

Increasing pole length from SS − 5 cm to SS + 10 cm during DP on a treadmill induced lower O_2_-cost and total vertical displacement of COM_z_, and the advantage of longer poles was greater in Moderate incline compared to Low incline conditions. The present study demonstrated how a change in pole length influences the O_2_-cost and kinematic of DP. Whether our findings also occur on snow and if pole lengths influence the overall performance need to be further elucidated. Our findings correspond with the temporary rule from FIS, which restricts the pole length to be longer than 83% of the body height in an attempt to reduce the use of DP during uphills in classic cross-country ski races.

## Electronic supplementary material

Below is the link to the electronic supplementary material.

Supplementary material 1 (AVI 4091 kb)

Supplementary material 2 (AVI 4363 kb)

## Abbreviations

ANOVA: Analysis of variance
COM: Center of mass
COM_z_: Vertical position of the center of mass
COM_zmin_: Minimum value of the vertical position of the center of mass
COM_zmax_: Maximum value of the vertical position of the center of mass
*D*_COM−pole plant_: The shortest distance between COM and the poles at pole plant
DIA: Diagonal stride; skiing technique which propulsive forces are transferred through poles and skies
DP: Double poling; skiing technique which propulsive forces are transferred sorely through the poles
SD: Standard deviation
SS: Self-selected
ES: Effect size
FIS: International Ski Federation
HF_max_: Maximal heart rate
Low incline: Low gradient uphill
Max: Maximum
Min: Minimum
Moderate incline: Moderate gradient uphill
O_2_-cost: Oxygen cost
ROM: Range of motion
RPE: Rate of perceived exertion
SS − 5 cm to SS + 10 cm: Increase from SS − 5 cm to SS to SS + 5 cm to SS + 10
